# 177Lutetium-PSMA-I&T therapy for metastatic castration-resistant prostate cancer (mCRPC): the first multicenter real-world study of 177Lu-PSMA-I&T in Brazil

**DOI:** 10.1186/s41824-026-00305-8

**Published:** 2026-06-09

**Authors:** Helena F. Bruzzi, Gabriela C. K. Lopes, Hannah B. V. Bekierman, Sergio Altino de Almeida, José Alexandre Pedrosa, Camila Mosci, Haonne S. Abboud, Christiane Magalhães, Isabella Palazzio, Laura Fernandes, Vinicius Freire da Silva, Juliana Tarouquella da Silva Andrade, Daniel Herchenhorn

**Affiliations:** 1 Instituto D’Or de Ensino e Pesquisa IDOR, Oncologia D’Or, Rio de Janeiro, Brazil; 2 Postgraduate Program, Central Coordination of Continuing Education (CCEC), PUC-Rio, Rio de Janeiro, Brazil; 3 Department of Nuclear Medicine, Rede D’Or São Luiz, Rio de Janeiro, Brazil; 4 Department of Urology, National Cancer Institute (INCA), Rio de Janeiro, Brazil; 5 Department of Nuclear Medicine, Rede D’Or São Luiz, São Paulo, Brazil; 6 Clínica São Vicente, Oncologia D’Or Rua Marques de São Vicente 52, 4o andar, Gávea, Rio de Janeiro, 22451-040 Brazil

**Keywords:** 177Lu-PSMA-I&T, Metastatic castration-resistant prostate cancer, Real-world evidence, Visceral metastasis

## Abstract

**Purpose:**

Despite the approval of 177Lu-PSMA-617 as standard treatment for patients with mCRPC, at least 50% of patients do not respond to the therapy, especially those with visceral disease. This study analyzes real-world outcomes of 177Lu-PSMA-I&T used in heavily pretreated patients in Brazil.

**Methods:**

Retrospective analysis of patients with mCRPC previously treated with at least one androgen receptor pathway inhibitor (ARPI) who underwent 177Lu-PSMA-I&T between 2020 and 2025 in two large oncology centers. Our primary endpoint was prostate-specific antigen (PSA) response rate of 50% or more (PSA50). Secondary endpoints included overall survival (OS) and time to next sequential therapies (TNST). Statistical analyses were performed in JAMOVI and RStudio.

**Results:**

Forty-three patients were included, with median age 74 years and median baseline PSA 41 ng/mL. Prior to 177Lu-PSMA-I&T, 86% received more than one ARPI line and 23.3% underwent more than one taxane-based chemotherapy. Visceral disease was present in 41.9%. The overall PSA50 response for all patients was 44.2% and for patients with visceral disease was 33.3%. Median OS was 13.9 months [95% confidence interval (CI) 10.9–19.2] and 12-month survival was 55.5% [95% CI 42.1%-73.3%]. Out of the 24 patients who received subsequent therapies, median TNST was 2.9 months [95% CI 1.6–5.4].

**Conclusions:**

Our results showed that 177Lu-PSMA-I&T achieved a PSA response comparable to those treated with 177Lu-PSMA-617 in randomized trials, despite our heavily treated patients’ characteristics. A significant number of patients had visceral disease with expected lower PSA response, highlighting the need for more active combinations in this subgroup.

**Supplementary Information:**

The online version contains supplementary material available at 10.1186/s41824-026-00305-8.

## Introduction

Metastatic castration-resistant prostate cancer (mCRPC) presents a formidable clinical challenge, and the recent approval of 177Lu-PSMA-617 (177Lu) by the Food and Drug Administration (FDA) followed by other international agencies represents a significant advancement in treatment and a new paradigm shift due to its unique mechanism of action. This therapy delivers targeted radiation to PSMA-expressing cells, offering precision while minimizing damage to normal tissues.

In the pivotal phase III VISION trial (Sartor et al. 2021), the addition of 177Lu to standard care significantly extended survival for patients with mCRPC and progressive disease, compared to the control arm, particularly in those who had previously received treatment with androgen receptor pathway inhibitors (ARPI) and taxanes. The treatment was associated with manageable toxic effects. Additionally, this radiopharmaceutical demonstrated benefits in other relevant secondary endpoints, prolonging the time to symptomatic skeletal events, delaying deterioration in health-related quality of life and pain, and postponing biochemical progression.

Another randomized phase II trial, the TheraP trial (Hofman et al. 2021), showcased promising efficacy and safety of 177Lu in men with mCRPC who had disease progression after multiple lines of therapy. Compared to cabazitaxel, TheraP demonstrated superior activity, safety, and patient-reported outcomes with 177Lu in patients progressing after docetaxel. These positive results have sparked interest in exploring rational treatment combinations and considering this treatment earlier in the course of prostate cancer, with ongoing trials combining it with androgen receptor pathway inhibitors (ARPI) (Emmett et al. 2024), poly (ADP-ribose) polymerase inhibitors (Sandhu et al. 2023), and immune checkpoint inhibitors (ICI) (Subramaniam et al. 2025). Furthermore, the upfront use of this radiopharmaceutical in men with newly diagnosed metastatic hormone-sensitive prostate cancer is also being investigated, and initial results of the PSMAfore and PSMAddition trials have also been presented, reinforcing its benefit in the pre-chemotherapy stage but also in men with hormone-sensitive disease (Morris et al. 2024, Tagawa et al. 2023).

Despite these encouraging findings, challenges persist; as responses occur in less than 50% of the patients, and most are transitory (Sartor et al. 2021). Recent studies highlight these disparities between resource-rich countries and LMICs, where widespread implementation remains limited by logistical and financial constraints, resource scarcity, and regulatory and educational barriers (Brink et al. 2025, Al-Ibraheem et al. 2025, Abdel-Wahab et al. 2024).

Although this therapy was recently approved in Brazil by Brazilian Health Regulatory Agency (Anvisa), access to new technologies is a challenge for low-middle-income countries, particularly in countries like Brazil, where access to 177Lu remains limited due to its high costs. Prior to the approval of 177Lu, 177Lu-PSMA-I&T (177Lu-I&T) was available in the country only in selected centers.

Ongoing research and real-world data are crucial to corroborate treatment efficacy and patient selection outside clinical trials, emphasizing the applicability of 177Lu-I&T in a broader clinical context and comparing its results with the recent standard approved pharmaceutical. This study addresses the existing gap in real-world evidence, utilizing retrospective data collected from reference Oncology Centers in Brazil to provide a comprehensive understanding of 177Lu-I&T therapy outcomes. By drawing parallels with randomized trials like TheraP and VISION, the data presented herein underscore the external validity of these trials, emphasizing the impact and effectiveness of radioligands in a broader clinical context.

Our work will also provide information on treatment selection and outcomes following progression after 177Lu-I&T, offering further insight into therapy sequencing for patients with disease progression after radionuclide therapy.

## Methods

### Patient selection

The study encompassed mCRPC patients treated between 2019 and 2025, from two reference oncology centers in Brazil (Oncologia D`Or Rio de Janeiro and Vila Nova Star Sao Paulo) that received at least one cycle of 177Lu-I&T. We selected adult patients with mCRPC, who had undergone previous treatment with one or more approved ARPIs and experienced disease progression. The eligibility criteria for participation in the study were designed to be inclusive, with no limit on the number of ARPI treatments, which allowed for a wide range of prior therapies to be included. In addition, patients who had undergone none to three prior taxane regimens were also included in the selection process. To minimize selection bias, we included all consecutive patients who meet the eligibility criteria. Exclusion criteria followed the VISION trial parameters, excluding patients who did not meet PSMA-positron emission tomography (PET) positivity requirements (at least one PSMA-positive lesion and no PSMA-negative lesions on screening exams pre-treatment) or had incomplete clinical registries. Data extraction was performed systematically and the cutoff date for data inclusion was 05/30/2025.

The planned range of 177Lu-PSMA-I&T cycles was up to 6, at 6 weeks intervals, with each cycle consisting of intravenous administration of 200 mCi (7.4GBq) of 177Lu-I&T under the supervision of the nuclear medicine team and of a multi-disciplinary uro-oncology tumor board.

### Imaging approach

In both centers, patient selection for 177Lu-I&T therapy followed the PSMA uptake criteria of the VISION trial, requiring PSMA-positive and no PSMA-negative lesions. Additionally at the Rio de Janeiro center, dual-tracer PET imaging with fluorodeoxyglucose (FDG) was additionally used to refine selection, allowing exclusion of lesions with discordant FDG-positive/PSMA-negative uptake (Iravani et al. 2020).

Imaging assessments were conducted at baseline and at regular intervals during treatment to monitor disease progression and response to therapy. Radiological reports were reviewed by a panel of experienced radiologists to ensure consistency and accuracy in interpreting the results. Laboratory tests and PSA measurements were obtained prior to each treatment cycle and six weeks after the cycle, following institutional monitoring protocols.

### Endpoints

The primary endpoint was prostate-specific antigen (PSA) response, defined as a ≥ 50% reduction from baseline (PSA50 response). PSA levels were measured prior to each 177Lu cycle and 4–6 weeks after completion. Best PSA response was considered as the lowest level achieved anytime during treatment and within 6 months from the last cycle.

Secondary outcomes included overall survival (OS), defined as the interval from the first 177Lu-PSMA-I&T cycle until death from any cause or date of last follow-up. Time to next sequential therapies (TNST), defined as the interval from the last 177Lu-I&T treatment cycle to the initiation of subsequent anticancer therapy after progression. Due to the retrospective nature of our study only grade 3–4 adverse events were captured per the Common Terminology Criteria for Adverse Events (CTCAE) version 5.0 and unacceptable toxicity leading to treatment discontinuation was also analyzed.

Further analyses included the exploration of outcomes for patients with visceral metastasis. Radiographic outcomes according to RECIST criteria as well as radiographic disease free-survival were not included due to the study´s retrospective design and variability in imaging practices across treatment centers.

### Statistical analysis

Survival outcomes were estimated using Kaplan-Meier method and compared with the log-rank test. Hazard ratios with 95% confidence intervals were calculated using Cox proportional hazards regression. The association between PSA response and visceral disease was assessed using Cox regression, including an interaction term.

Results were considered statistically significant if p-value < 0.05. Analyses were performed using JAMOVI (v2.6.26.0) and RStudio (v2025.09.2 + 418) platforms.

### Ethics approval and consent to participate

This study protocol was approved by the Instituto D’Or de Pesquisa e Ensino review board (protocol number: 7.335.537) and by Plataforma Brazil (CAAE: 83791424.1.0000.5249). The requirement for written informed consent was waived by the Instituto D’Or de Pesquisa e Ensino ethics committee due to the retrospective and non-interventional nature of the study.

This observational study was reported according to STROBE (Strengthening the Reporting of Observational Studies in Epidemiology) guidelines.

## Results

**Fig. 1:**
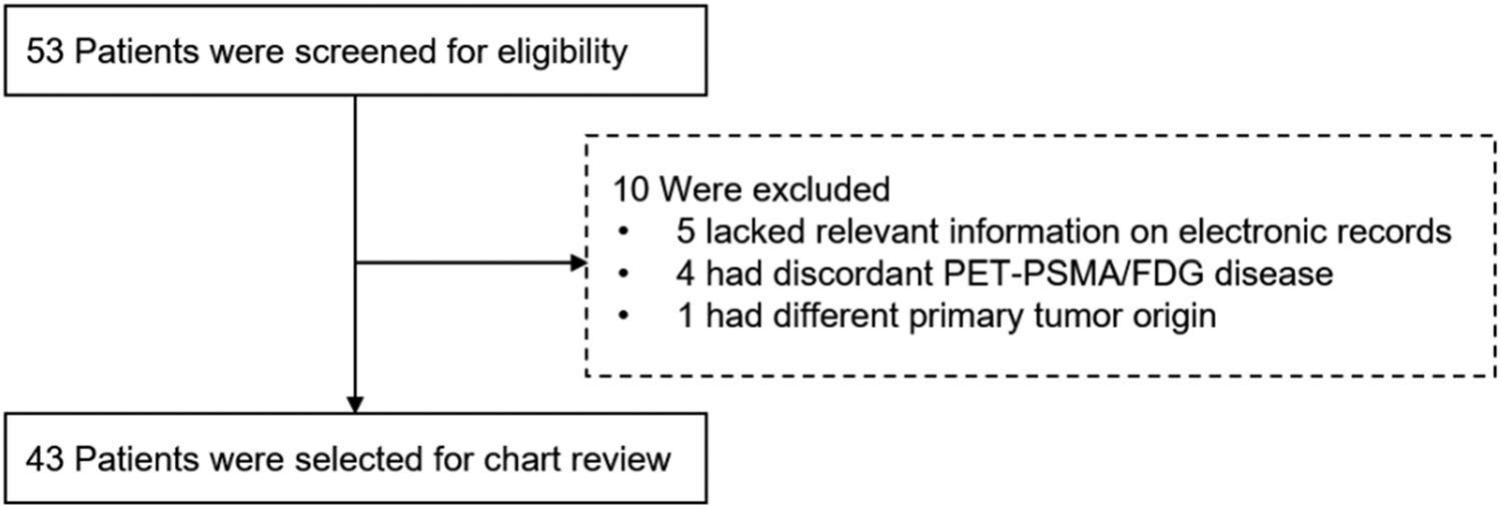
Flowchart of patient selection. PET = positron emission tomography. FDG = fluorodeoxyglucose

Initial screening identified 53 patients who underwent the first 177Lu-PSMA-I&T therapy from 2019 to 2025, before approval and reimbursement of 177Lu-PSMA-617 (Fig. 1).

A total of 43 patients diagnosed with mCRPC were included in the final report; 24 were from the Rio de Janeiro center and 19 from the São Paulo center.

From the 24 in Rio de Janeiro center who underwent a FDG-PET in the baseline screening, 19 had available exam results for review, which showed: 63.2% had high PSMA and low FDG expression, 26.3% high PSMA and high FDG, and 10.5% high PSMA with no FDG uptake (Iravani et al. 2020). FDG-PET data was unavailable for the remaining five patients from this center.

The median age of the entire patient cohort was 74 years (range: 49–92). Visceral disease was observed in 41.9% (18/43) of patients. The most frequently involved organs were the lungs (23.3%), liver (14%), and central nervous system (2.3%). Additional visceral metastatic sites were observed in 14% of patients, including the adrenal glands (*n* = 3) and peritoneum (*n* = 3). A total of 23 metastatic sites were identified, with some patients with multiple organs involvement. Table 1 summarizes the baseline characteristics of the patient’s cohort.

To categorize the pattern of bone involvement in our cohort, we used the proposed classification by Eiber et al. (2018), which created a standardized language for image reporting with PSMA-ligand PET/computed tomography (CT) based on molecular imaging TNM system.

Among the 43 included patients we stratified 39 between the four subcategories of bone disease: 28 (71.8%) had disseminated involvement; 8 (20.5%) had oligometastatic disease; 2 (5.1%) had diffuse marrow involvement; and 1 had unifocal involvement.

Median baseline PSA previous to 177Lu-PSMA-I&T therapy was 41 ng/mL (0.046–2109) and 55.8% had Gleason of 8 or above.

Analysis of prior treatment exposure showed that 86% of patients received more than one line of androgen receptor pathway inhibitor (ARPI) therapy. In addition, 67.4% of patients had been treated with at least one taxane-based chemotherapy regimen, of whom 23.3% received two or more taxane lines. Overall, 14 patients (32.5%) had not received any prior taxane-based chemotherapy (were deemed unfit for chemotherapy or refused it).

**Table 1:** Baseline patient characteristics

Characteristic	All Patients (*N* = 43)
Median age (range) - yr	74 (49–92)
Gleason score at diagnosis - no (%)	
5–7	11 (25.6)
8–9	24 (55.8)
Unknown	8 (18.6)
Median PSA level (range) - ng/ml	41 (0.046–2109)
Metastatic sites - no. (%) ^a^	
Bone only	7 (16.3)
Lymph node only	1 (2.3)
Bone and lymph node	15 (34.9)
Visceral disease details - no. (%)	
Lung	10 (23.3)
Liver	6 (14)
CNS	1 (2.3)
Other ^b^	6 (14)
Previous prostatectomy - no. (%)	29 (67.4)
Previous ARPI- no. (%)	
One regimen	6 (14)
Two regimens	24 (55.8)
More than two regimens	13 (30.2)
Previous taxane therapy - no. (%)	
None ^c^	14 (32.5)
One regimen	19 (44.2)
Two regimens	7 (16.3)
More than two regimens	3 (7)

PSA = Prostate Specific Antigen. ARPI = Androgen receptor pathway inhibitor

^a^ Patients may have more than one metastatic site; percentages may not sum to 100%

^b^ Patients with metastasis to the adrenal glands or peritoneum

^c^ Refused or were not fit for chemotherapy

The median number of cycles administered was four (range 1–6). Reasons for treatment discontinuation were disease progression in 62.8%, unacceptable toxicity in 11.6%, and completion of planned cycles in 25.6% (11 patients).

**Fig. 2:**
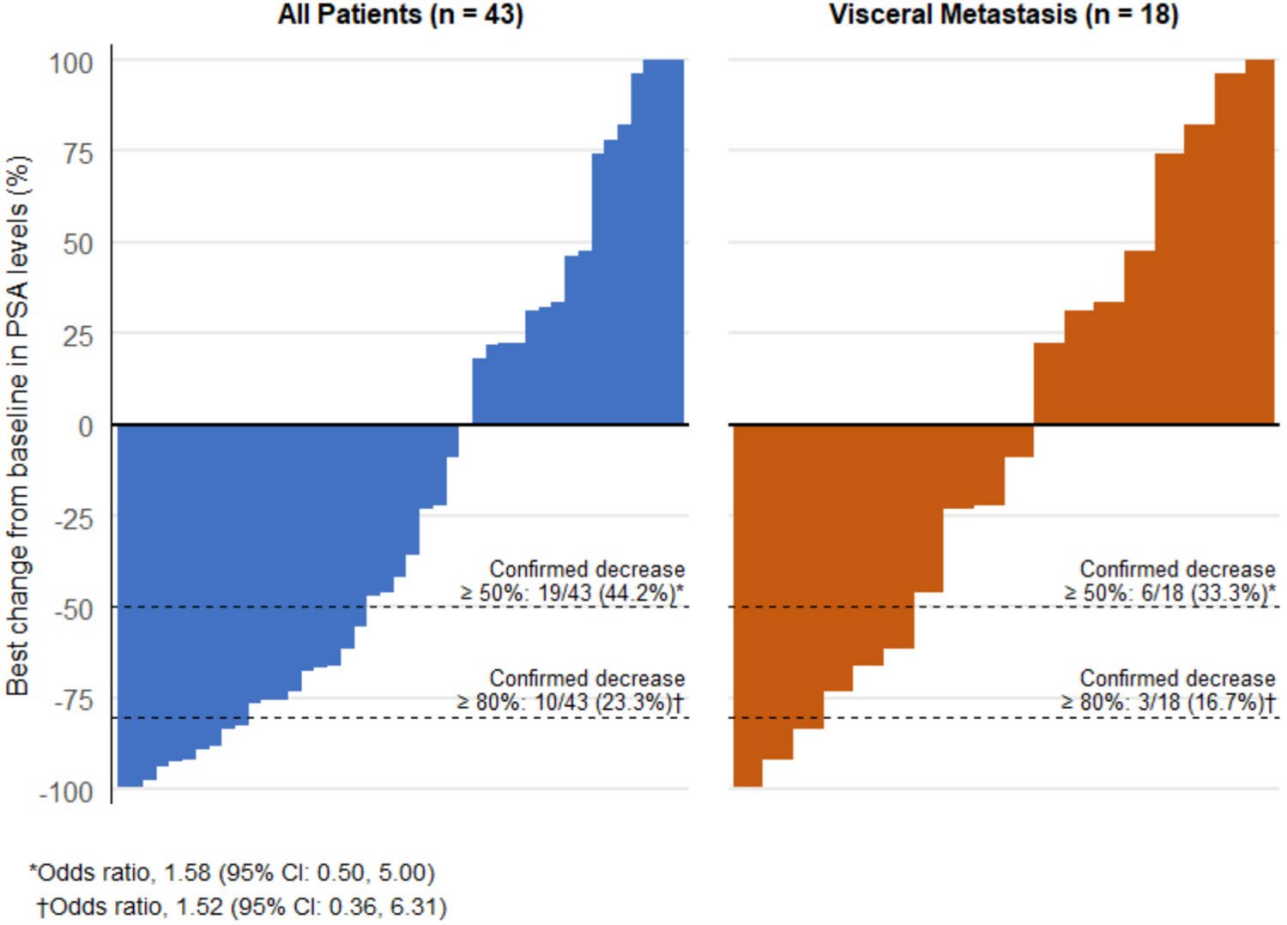
Waterfall plot of prostate specific antigen responses to 177Lu-PSMA-I&T. The proportion of patients with any decrease in best percentage change from baseline was 60.5% (26/43) in all patients and 55.6% (10/18) in the visceral metastasis subgroup. Increases greater than 100% were limited to 100%. Patients with persistently undetectable PSA (< 0.2 ng/mL) were assigned 0% change (*n* = 1). PSA = prostate specific antigen. 177Lu-PSMA-I&T = Lutetium-177-labeled prostate-specific membrane antigen inhibitor and theragnostic

PSA50 response was achieved in 44.2% of patients (19/43), and PSA80 response (≥ 80% PSA decline) in 23.3% as seen in Fig. 2. Achievement of undetectable PSA levels (< 0.2 ng/mL) occurred in only 9.3% of patients. Among these, 1 patient achieved undetectable PSA from detectable baseline levels, while 1 patient maintained persistently undetectable PSA. Baseline characteristics according to PSA50 response are shown in Table 2.

Among patients with visceral disease (18/43), 33.3% achieved a PSA50 response and 16.7% a PSA response of ≥ 80%. Furthermore, two patients reached undetectable PSA from baseline levels.

**Table 2:** Baseline patient characteristics according to PSA response status

Characteristic	PSA Response < 50% (*N* = 24)	PSA Response ≥ 50% (*N* = 19)
Median age (range) - yr	69 (49–92)	76 (53–92)
Gleason score at diagnosis - no (%)		
5–7	3 (12.5)	8 (42.1)
8–9	14 (58.3)	10 (52.6)
Unknown	7 (29.2)	1 (5.3)
Median PSA level (range) - ng/mL	29.4 (0.046-713)	51.6 (0.06–2109)
Metastatic sites - no. (%) ^a^		
Bone only	4 (16.7)	3 (15.8)
Lymph node only	0 (0)	1 (5.3)
Bone and lymph node	6 (25)	9 (47.4)
Visceral disease details - no. (%)		
Lung	6 (25)	4 (21.1)
Liver	4 (16.7)	2 (10.5)
CNS	1 (4.2)	0 (0)
Other ^b^	6 (25)	0 (0)
Previous prostatectomy - no. (%)	15 (62.5)	14 (73.7)
Previous ARPI- no. (%)		
One regimen	3 (12.5)	3 (15.8)
Two regimens	12 (50.0)	12 (63.1)
More than two regimens	9 (37.5)	4 (21.1)
Previous taxane therapy - no. (%)		
None ^c^	6 (25.0)	8 (42.1)
One regimen	10 (41.7)	9 (47.3)
Two regimens	6 (25.0)	1 (5.3)
More than two regimens	2 (8.3)	1 (5.3)

PSA = Prostate Specific Antigen. ARPI = Androgen receptor pathway inhibitor

^a^ Patients may have more than one metastatic site; percentages may not sum to 100%

^b^ Patients with metastasis to the adrenal glands or peritoneum

^c^ Refused or were not fit for chemotherapy

**Fig. 3:**
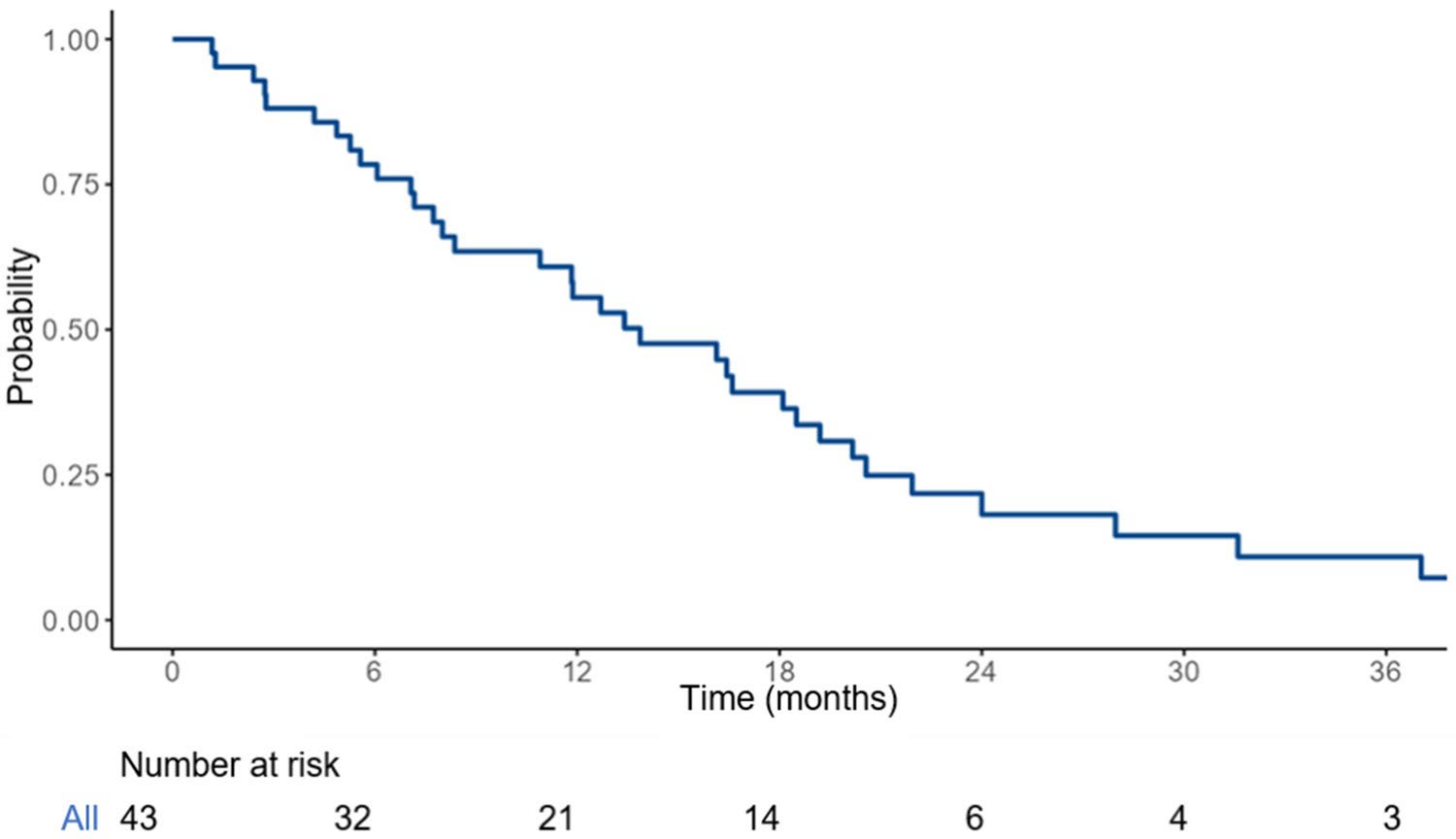
Overall survival of the entire cohort (*n* = 43). The number of patients at risk at each time point is shown below the figure

The median follow-up is 11.9 months (1.2–66.5 months). Median OS was 13.9 months [95% CI 10.9–19.2] and the estimated 12 and 24-month survival was 55.5% [95% CI 42.1–73.3] and 18.1% [95% CI 8.8–37.2], respectively (Fig. 3).

**Fig. 4:**
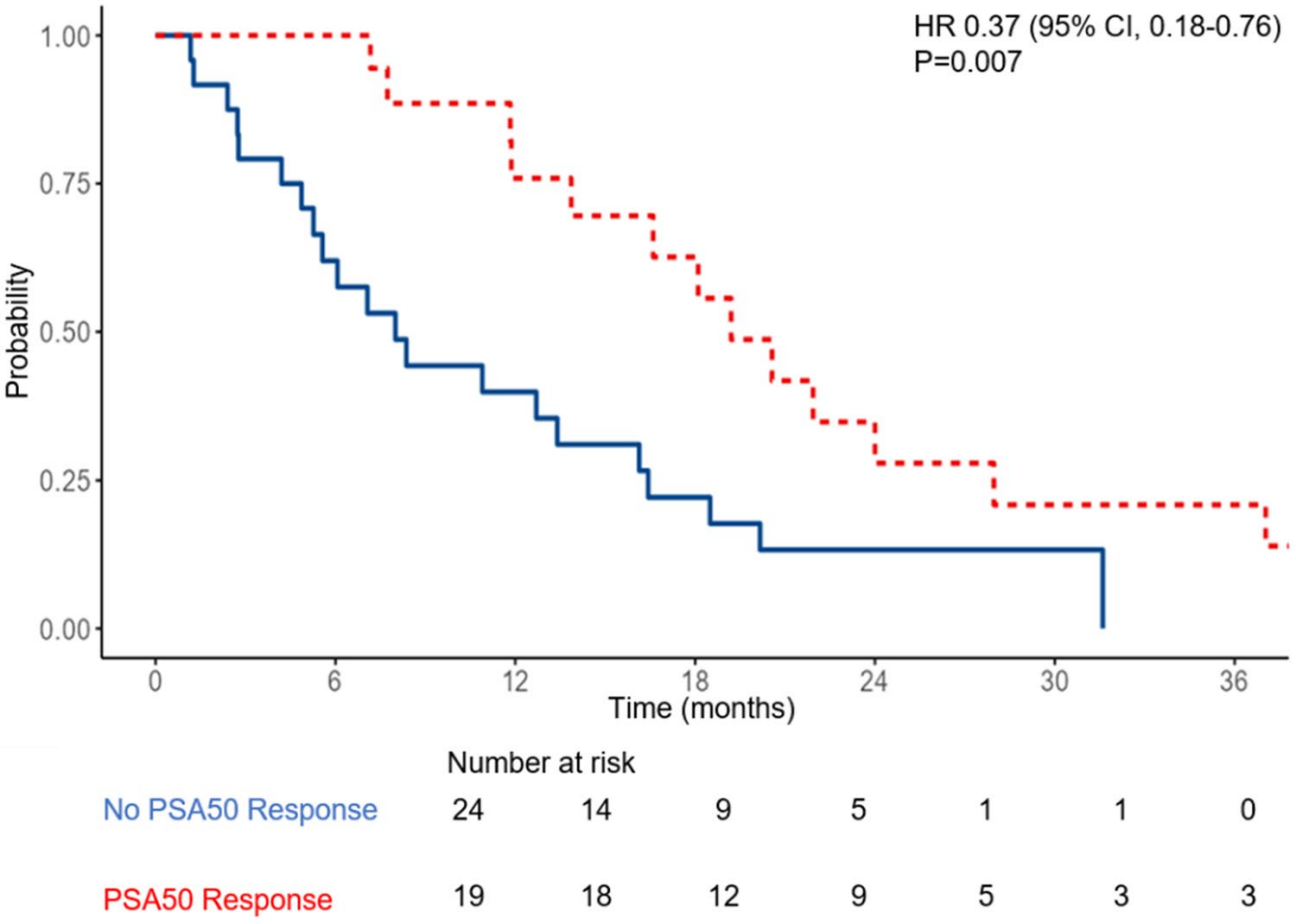
Survival according to PSA response status. The number of patients at risk at each time point is shown below the figure. PSA = prostate-specific antigen. PSA50 = ≥ 50% PSA decline. HR = hazard ratio. CI = confidence interval

Biochemical responders (PSA decline ≥ 50%) had a longer median OS of 19.2 months [95% CI 16.6-Not reached] and the estimated 12-month survival was 75.9% [95% CI 57.8–99.7], while those who did not achieve a PSA50 response had an estimated 12-month survival of 39.84% [95% CI 24.1–65.9] (Fig. 4).

**Fig. 5:**
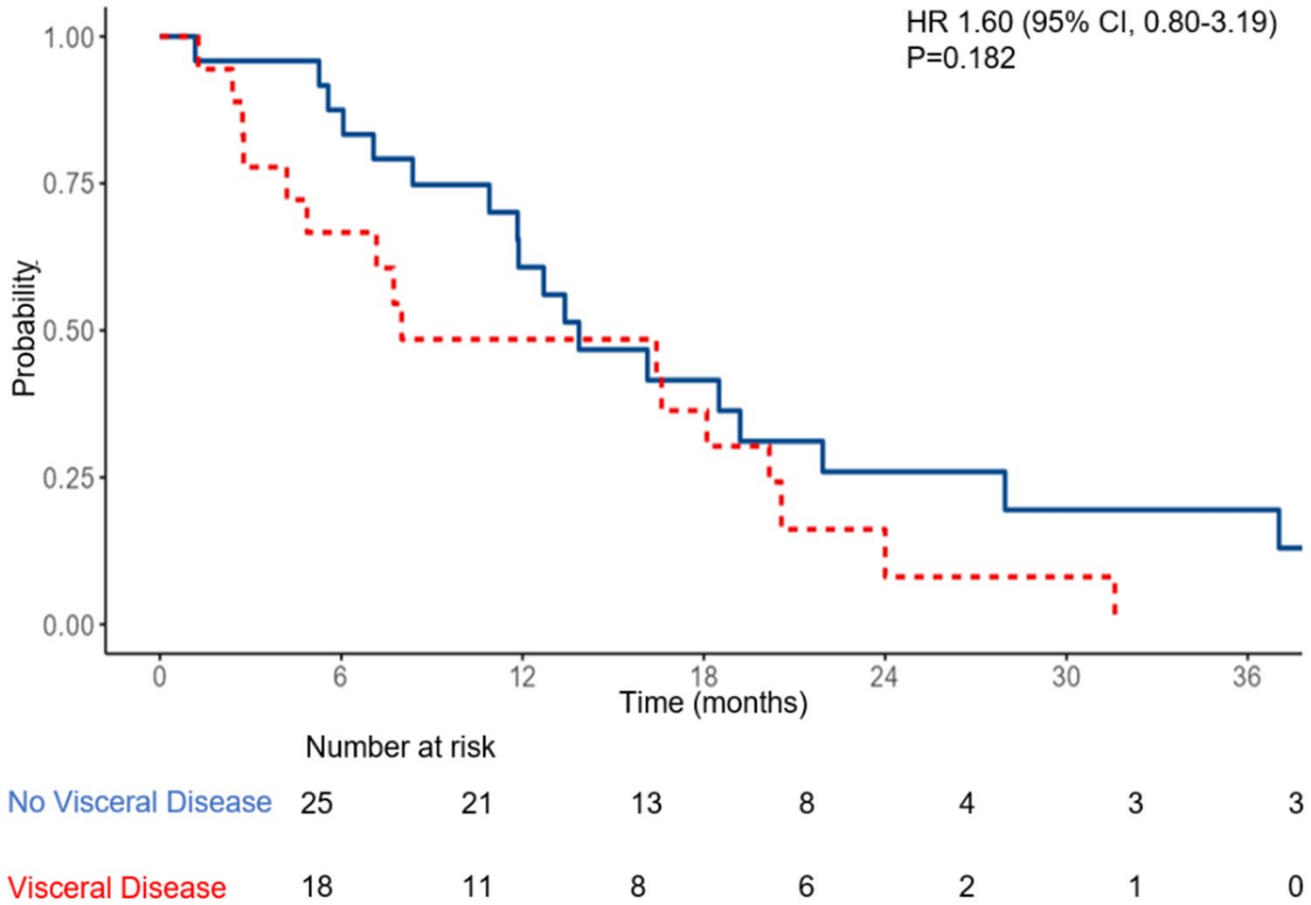
Survival according to visceral disease status. The number of patients at risk at each time point is shown below the figure. HR = hazard ratio. CI = confidence interval

Patients without visceral disease had a median OS of 13.9 months [95% CI 11.8–28] and the estimated 12-month survival was 60.7% [95% CI 43.6–84.7]. For patients with visceral disease, the median OS was 8 months [95% CI 4.9–24], while the 12-month survival was 48.5% [95% CI 29.8–79]. However, due to the small sample size (only 8 patients with visceral disease at risk in the 12-month timepoint) there is a wide confidence interval which warrants caution on interpretation of the results (Fig. 5).

**Fig. 6:**
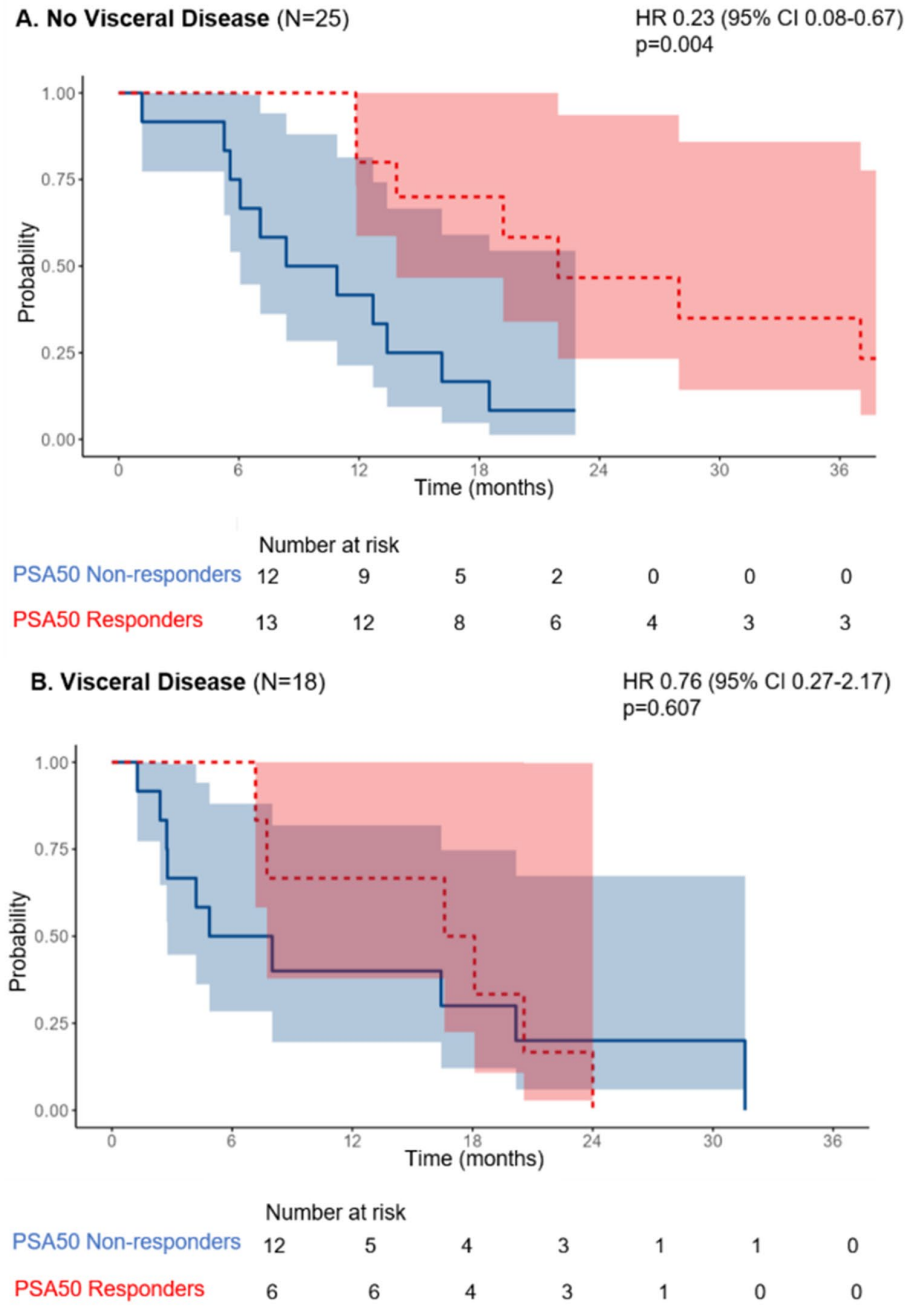
Overall survival (OS) according to the presence of visceral disease and PSA50 Response. The graphic in panel **A** shows OS in patients without visceral disease (25/43), while the one in panel **B** shows OS for patients who had visceral disease. Shading represents the 95% confidence interval for survival probability. The number of patients at risk at each time point is shown below the figure. PSA = prostate-specific antigen. PSA50 = ≥ 50% PSA decline. HR = hazard ratio. CI = confidence interval

The association between PSA50 response and OS was stratified by the presence of visceral disease and results are described in Table 3 and depicted in Fig. 6. In patients without visceral disease, the responders had a statistically significant (p-value = 0.004) reduction in the risk of death compared to non-responders with a Hazard Ratio of 0.23 (95% CI: 0.08–0.67) and OS improvement of 12.3 months. In contrast, the difference observed in patients with visceral disease did not reach statistical significance, probably a reflection of the small group of responders (6/18).

Furthermore, the interaction analysis between PSA50 response and visceral disease status was not statistically significant (p-value = 0.120), which likely reflects the small sample size and requires caution upon interpretation of the results.

**Table 3:** Overall survival stratified by PSA50 response and visceral disease

Characteristic	Visceral Disease (*N* = 18)	No Visceral Disease (*N* = 25)
**PSA50 Responders**,** n (%)**	6 (33.3)	13 (52)
Median OS, months	17.4	21.9
12-month OS rate, %	66.7	80
Death, n (%)	6 (100)	7 (53.8)
**PSA50 Non-responders**,** n (%)**	12 (66.7)	12 (48)
Median OS, months	6.4	9.6
12-month OS rate, %	40	41.7
Death, n (%)	10 (83.3)	11 (91.7)
**Statistical Comparison**		
HR (responders vs. non-responders)	0.76 [0.27–2.17]	0.23 [0.08–0.67]
Log-rank p-value	0.607	0.004

HR = Hazard Ratio. CI = confidence interval. OS = overall survival. PSA50 = prostate-specific antigen decline ≥ 50% from baseline

**Fig. 7:**
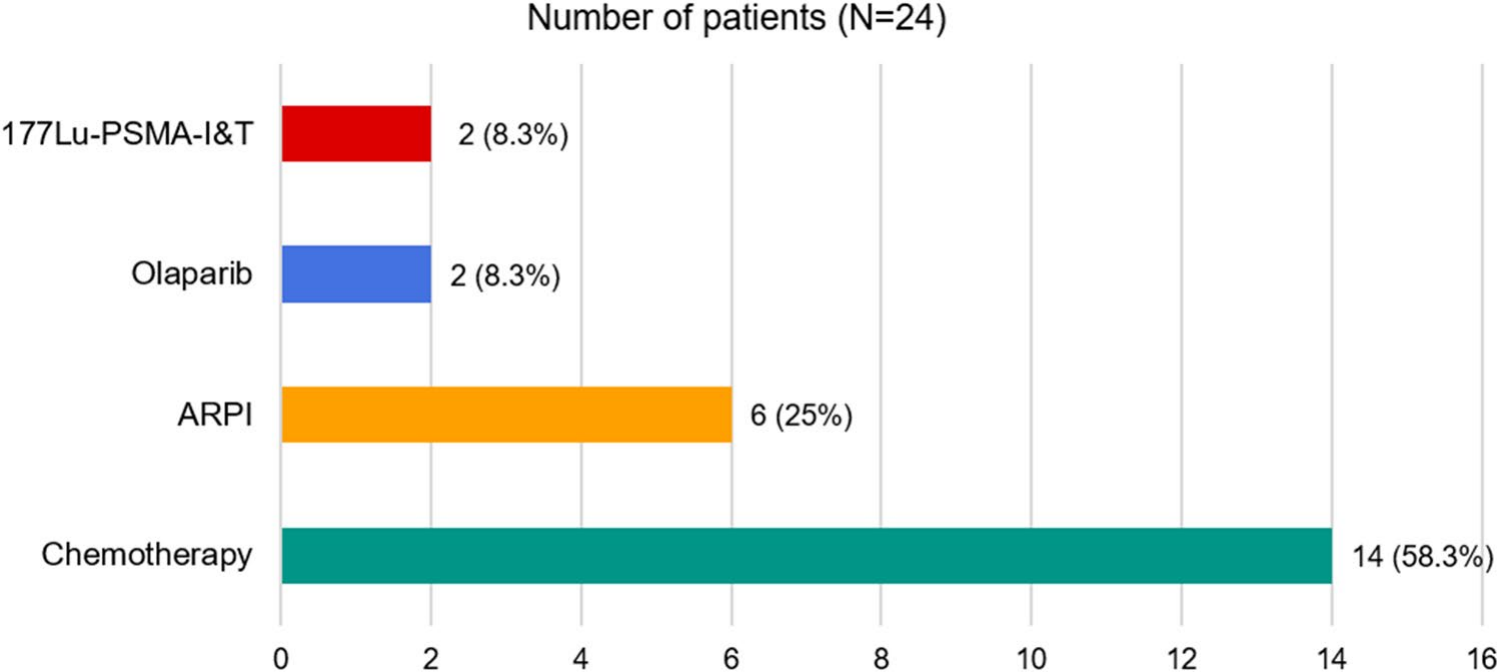
Subsequent therapies after 177Lu-PSMA-I&T. ARPI = Androgen receptor pathway inhibitor

Subsequent therapies were received by 24 (55.8%) patients, 58.3% of them underwent additional chemotherapy, 25% received another ARPI, 8.3% received Olaparib and 8.3% were re-treated with 177-Lu as described in Fig. 7. Median time to next sequential therapy (TNST) in this group of patients was only 2.9 months [95% CI 1.6–5.4].

Furthermore, grade 3 or 4 adverse events were seen in only 16.3% (seven patients). Hematologic adverse events observed included severe thrombocytopenia (2), pancytopenia (1), and myelodysplastic syndrome (1). Pneumonitis was observed in one patient. In addition, one patient in our cohort who had metastasis to the central nervous system (CNS) and another who had bone metastasis to the skull base developed cerebral edema, although the mechanism by which this occurs is not yet well defined, this effect may be secondary to Lutetium crossing the blood-brain barrier. Both cases were resistant to corticosteroid therapy and required treatment discontinuation for this reason.

## Discussion

177Lu-PSMA therapy emerged as a promising therapeutic option for patients with mCRPC, with trials demonstrating survival benefit and mild associated toxicities in comparison with standard treatment options (Sartor et al. 2021, Hofman et al. 2021). However, optimal criteria for patient selection and the timing of therapy application across the disease trajectory remain to be defined. Real-world data may provide valuable insights to better align future research with clinical practice.

Our study is the first multicenter real-world analysis to investigate 177Lu-PSMA-I&T outcomes in Brazil, where accessibility to newer treatments is a challenge.

Originally, PSMA radioligand therapies—including PSMA-I&T—were developed through academic and research collaborations, with early preclinical and clinical work conducted in Europe; PSMA-I&T was synthesized by linking the PSMA-targeting ligand to an appropriate chelator and labeling it with Lu177 for therapeutic use. These developments built on foundational work in PSMA targeting aimed at creating effective theranostic agents for mCRPC.

The population of our study was heavily pretreated, with 86% of patients receiving two or more previous ARPIs, compared with 44.7% of patients in the Vision trial and only 21% of patients in the TheraP trial who received both enzalutamide and abiraterone (Sartor et al. 2021, Hofman et al. 2021). In addition, 24% of patients received more than one line of chemotherapy, further highlighting that this cohort extended beyond the populations typically represented in randomized clinical trials.

A meta-analysis by Kim et al. (Kim and Kim 2018), evaluated the therapeutic responses after the first cycle of 177Lu-PSMA-617 radioligand therapy with a total of 455 patients with mCRPC included and found that any PSA decline after the first cycle was associated with better survival. Our study demonstrated a PSA50 response in 44.2% of patients, which is comparable to that reported in the Vision and TheraP trials: 66% PSA50 response at the TheraP trial; and 46% in the VISION trial. This highlights the effectiveness of 177Lu-PSMA-I&T as a treatment option for mCRPC in real-world scenarios, with consistency of outcomes showing the reliability and robustness of this treatment approach even in a non-selected population enriched with visceral metastasis.

The TheraP trial protocol established sufficient PSMA avidity as an inclusion criterion to enhance patient selection. In our study, 24 patients from the Rio de Janeiro center were selected using the same TheraP criteria and achieved a median OS of 16.1 months [95% CI 10.9–19.2], consistent with the 16.4 months reported in TheraP. The remaining 19 patients from São Paulo, selected using VISION criteria, had a median OS of 13.4 months. While this difference was not statistically significant (*p* = 0.759), it suggests that stricter patient selection may influence outcomes, though our study is not powered to perform a formal comparison between selection strategies.

Among our entire cohort (*n* = 43), median OS was 13.9 months [95% CI 10.9–19.2], which likely reflects this heterogeneity in selection approaches. The VISION trial selection criteria considered eligible patients with mCRPC and at least one PSMA-positive metastatic lesion and no PSMA-negative lesions and found a median overall survival of 15.3 months, which, although not comparable, is slightly higher than our analysis. Other real-world clinical experiences have reported varying results, further emphasizing the importance of defining patient selection and treatment protocols (Ling et al. 2025).

A recently published real-world study from the Netherlands, with 177Lu-PSMA-I&T, a similar number of patients and rate of lung and liver involvement (liver 14% vs. 14%, lung 23% vs. 12%) showed lower efficacy compared to our outcomes, including a PSA50 response of 16% versus 44.2% and median OS of 8.1 versus 13.9 months. Despite a comparable rate of patients who had two or more ARPI lines between studies (84% versus 86%), 88% of their cohort received prior cabazitaxel, while 23.3% of our cohort underwent two or more taxane-based regimens, which likely explains their worse response. Additionally, they received a lower median number of treatment cycles (2 versus 4), and their PET selection criteria followed those of the VISION trial, whereas our Rio de Janeiro center followed TheraP`s stricter criteria (Ling et al. 2025).

A more profound understanding of the variables that can influence response can improve patient selection. Both VISION and TheraP trials have established 68-Ga-PSMA-11 standardized uptake value (SUV) mean values as prognostic for overall survival. Gafita et al. (2021) in their nomogram study validated (SUV) mean for outcomes prediction in 177Lu-PSMA-I&T. Accordingly, Muniz et al. (2024) in a retrospective study, showed (SUV) mean to be associated with longer survival for those with a baseline liver SUVmean of 8 or higher in patients with liver metastases.

In the VISION trial, a subgroup of patients with visceral disease are reported to have an expected worse prognosis, an observation supported by published retrospective studies, but a more comprehensive analysis of this subgroup’s specific parameters and outcomes was not performed (Muniz et al. 2024, Kalender et al. 2025, Khreish et al. 2021).

Most of the available data on the use of 177Lu-PSMA-617 for patients with mCRPC and visceral metastasis are found in retrospective analysis and real-world data, limiting the generalizability of findings. However, as this represents a subset of patients who have limited responses to standard therapies, it is fundamental to explore the potential of new and more effective therapeutic options.

Khreish et al. (2021) retrospectively analyzed 28 patients with mCRPC with liver metastasis and evaluated response by modified PET response criteria in solid tumors. Three factors were associated with better OS: biochemical partial response after 2 courses of therapy, hepatic disease control (Complete Response, Partial Response and Stable Disease) and patient’s performance status. Similarly, we found an association between PSA50 response and OS in patients with visceral disease with a hazard ratio for death of HR: 0.76 [95% CI 0.27–2.17] relative to non-responders. Despite this difference, statistical significance was not reached, likely due to limited statistical power since there were only 6 PSA50 responders in the visceral disease subgroup.

Muniz et al. (2024) used a database of patients receiving 177Lu-PSMA-617 to look further into its efficacy in patients with mCRPC and liver metastases (43 patients). They found that the presence of liver metastasis was independently associated with shorter OS.

The PSA50 response rate and OS of patients with liver metastasis in the study were 30.2% (13/43) and 8.4 months, respectively. Comparable to the 33.3% (2/6) PSA50 response and 8 months OS found in our study. Furthermore, it is notable that these two responding patients also achieved a PSA80 response. Despite the overall poorer outcomes of those with liver metastasis, these results highlight how some may still have clinical benefit with 177Lu-PSMA therapy.

In addition, there was no clear difference in survival when stratifying by number of hepatic lesions. Khreish observed that liver tumor burden had no relationship with treatment response. These findings support the benefit of 177Lu-PSMA for patients even with large hepatic disease volume.

In our cohort, three patients underwent 177Lu-PSMA-I&T rechallenge after progression. Two of them achieved a PSA50 response during initial treatment, but responses to the re-exposition were poor. This cannot be extrapolated to definitive conclusions as the number of patients is low. In comparison, Rosar et al. (2024) showed a meaningful PSA response rate after the first rechallenge in a cohort of 47 patients previously exposed to 177Lu-PSMA-617, besides demonstrating a tendency to maintain response with subsequent rechallenges with no significant added toxicity.

Research is ongoing on combining 177Lu-PSMA-617 with other treatments in earlier disease states (Emmett et al. 2024, Sandhu et al. 2023, Subramaniam et al. 2025, Morris et al. 2024, Tagawa et al. 2023). The ENZA-p trial demonstrated improved PSA progression-free survival by adding [177Lu] Lu-PSMA-617 to enzalutamide in high-risk mCRPC patients (Emmett et al. 2024), while LuPARP showed promising activity for the combining of Olaparib with 177Lu-PSMA-617 (Sandhu et al. 2023). ICI combinations have also gained traction, demonstrating improved outcomes (Subramaniam et al. 2025, Sandhu et al. 2022, Aggarwal et al. 2023). Responses have been observed across different PSMA expression levels, including low-PSMA and visceral lesions (Aggarwal et al. 2023), supporting further research for broader mCRPC populations.

Another focus of research has been to explore biomarkers for better treatment selection. Recent data from Jani et al. (2025) correlated differences in circulating tumor DNA (ctDNA) profiles, indicating potential treatment resistance mechanisms with a focus on homologous recombination repair (HRR) gene alterations. A retrospective analysis using a large US database identified that AR and TSG alterations were associated with worse outcomes to 177Lu-PSMA-617 in patients with metastatic prostate cancer, while FOXA1 and NF1 alterations were linked to improved responses and TSG related to poorer responders (Panian et al. 2025). These results further underscore the importance of expanding genomic analysis for directing therapy sequences and providing the best outcome for patients. Unfortunately, such data was limited in most of our patients, which affected its reporting.

Finally, an economic perspective, although not within the scope of our trial, is relevant because 177Lu-PSMA-I&T is associated with high direct treatment costs per patient. For example, cost modeling in Dutch hospitals estimated that per-patient costs for 177Lu-PSMA-I&T therapy range from approximately €35,866 to €47,546 per treatment period, depending on the dosing regimen used, and reimbursement levels may not fully cover these costs, potentially leaving hospitals to absorb several thousand euros per patient. The widespread integration of 177Lu-PSMA-617 into contemporary treatment paradigms and international guidelines has even higher significant economic implications, reinforcing the need for evidence-based patient selection and rational sequencing within therapeutic pathways.

## Limitations

It is crucial to recognize the limitations of this study, such as the small sample size, which limited the statistical power for subgroup analyses, particularly for comparing outcomes between patient selection strategies and among patients with visceral metastases. The retrospective nature of our study resulted in incomplete data collection; with important prognostic variables such as performance status and grade 1–2 adverse events not being systematically recorded. Molecular characterization was not available in most patients, limiting our ability to identify possible biomarkers of response and was not included in the analysis. Additionally, we recognize that the lack of uniform radiographic outcomes due to interobserver variabilities among assistant physicians may impair the validation of response. SUVmean data collection was also limited for our initial analyses. Despite these limitations, this study provides relevant real-world clinical evidence on 177Lu-PSMA-I&T efficacy in a heavily pretreated population with a high prevalence of visceral disease that outlines information provided by Vision and TheraP trials.

## Conclusion

This study is the first multicenter real-world analysis of 177Lu-PSMA-I&T conducted in Brazil. Real-world efficacy of Lu-PSMA for patients with mCRPC in this Brazilian cohort was comparable to those achieved in randomized trials, especially due to the parallel PSA response rates and survival, despite patients being overall older and more extensively pretreated. At least half of the patients were able to receive subsequent therapies and toxicity was mild. Furthermore, our study included a significant number of patients with visceral disease with lower PSA response, which calls for the investigation of innovative combination therapies to target this vulnerable subgroup of patients.

## Supplementary Information

Below is the link to the electronic supplementary material.

Supplementary Material 1

## Data Availability

The datasets generated and analyzed for the development of the study are available from the corresponding author on reasonable request.
