# Supplementary material for: 177Lutetium-PSMA-I&T therapy for metastatic castration-resistant prostate cancer (mCRPC): the first multicenter real-world study of 177Lu-PSMA-I&T in Brazil
Source: EJNMMI Rep. 2026 Jun 9;10(1):22. doi: 10.1186/s41824-026-00305-8 (PMC13246990; doi:10.1186/s41824-026-00305-8)
Supplement: Supplementary file 1 — Supplementary Material 1 [file 41824_2026_305_MOESM1_ESM.docx]

**Table** Exploratory analysis of outcomes according to patient imaging-based selection criteria for the Rio de Janeiro (TheraP criteria) and São Paulo (VISION criteria) centers. Statistical association was calculated using Fisher's exact test for PSA50 response and Log-rank test for median overall survival. PSA = prostate-specific antigen. PSA50 = ≥50% PSA decline. OS = overall survival. CI = confidence interval

| **Outcomes** | **Rio de Janeiro** (N=24) | **São Paulo**  (N=19) | **p-value** |
| --- | --- | --- | --- |
| PSA50 response, n (%) | 11 (45.8) | 8 (42.1) | 1.000 |
| Median OS, months (95% CI) | 16.1 (10.9-19.2) | 13.4 (4.9-NotReached) | 0.759 |
| Hazard ratio (95% CI) | Reference | 0.90 (0.45-1.80) |  |
